# Supplementary material for: Extracellular Vesicles Derived from Mesenchymal Stromal Cells Delivered during Hypothermic Oxygenated Machine Perfusion Repair Ischemic/Reperfusion Damage of Kidneys from Extended Criteria Donors
Source: Biology (Basel). 2022 Feb 22;11(3):350. doi: 10.3390/biology11030350 (PMC8945029; doi:10.3390/biology11030350)
Supplement: Supplementary file 1 [file biology-11-00350-s001.zip › biology-1584954-supplementary.pdf]

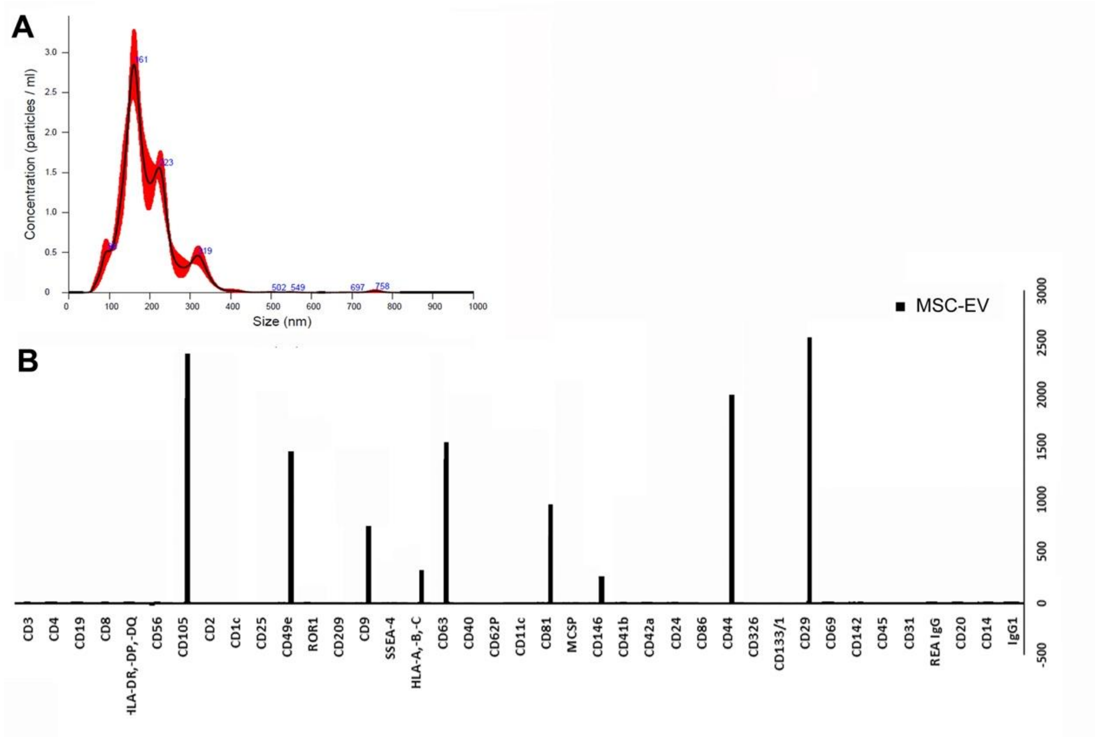

Figure S1. Characterization of EVs. (A) Representative graphs of nanoparticle tracking analysis showing size distribution of EVs. (B) Multiplex bead-based flow cytometry assay was used to detect EV surface signature. The graph shows a quantification of the median APC fluorescence values for all bead populations after background correction (medium control values subtracted from measured EVs values) of a representative EV preparation.
